# Novel Oncolytic Herpes Simplex Virus 1 VC2 Promotes Long-Lasting, Systemic Anti-melanoma Tumor Immune Responses and Increased Survival in an Immunocompetent B16F10-Derived Mouse Melanoma Model

**DOI:** 10.1128/JVI.01359-20

**Published:** 2021-01-13

**Authors:** Ifeanyi Kingsley Uche, Natalie Fowlkes, Luan Vu, Tatiane Watanabe, Mariano Carossino, Rafiq Nabi, Fabio del Piero, Jared S. Rudd, Konstantin G. Kousoulas, Paul J. F. Rider

**Affiliations:** a Division of Biotechnology and Molecular Medicine, Department of Pathobiological Sciences, School of Veterinary Medicine, Louisiana State University, Baton Rouge, Louisiana, USA; b Department of Pathobiological Sciences, School of Veterinary Medicine, Louisiana State University, Baton Rouge, Louisiana, USA; c Department of Biological Sciences, Louisiana State University, Baton Rouge, Louisiana, USA; d Pennington Biomedical Research Center, Baton Rouge, Louisiana, USA; e Louisiana Animal Diagnostic Laboratory, School of Veterinary Medicine, Louisiana State University, Baton Rouge, Louisiana, USA; Northwestern University

**Keywords:** B16F10, HSV-1, herpesvirus, immunotherapy, VC2, melanoma, oncolytic virotherapy, oncolytic viruses

## Abstract

Oncolytic virotherapy (OVT) is now understood to be an immunotherapy that uses viral infection to liberate tumor antigens in an immunogenic context to promote the development of antitumor immune responses. The only currently FDA-approved oncolytic virotherapy, T-Vec, is a modified type 1 herpes simplex virus (HSV-1). While T-Vec is associated with limited response rates, its modest efficacy supports the continued development of novel OVT viruses. Herein, we test the efficacy of a recombinant HSV-1, VC2, as an OVT in a syngeneic B16F10-derived mouse model of melanoma. VC2 possesses mutations that block its ability to enter neurons via axonal termini. This greatly enhances its safety profile by precluding the ability of the virus to establish latent infection. VC2 has been shown to be a safe, effective vaccine against both HSV-1 and HSV-2 infection in mice, guinea pigs, and nonhuman primates. We found that VC2 slows tumor growth rates and that VC2 treatment significantly enhances survival of tumor-engrafted, VC2-treated mice over control treatments. VC2-treated mice that survived initial tumor engraftment were resistant to a second engraftment as well as colonization of lungs by intravenous introduction of tumor cells. We found that VC2 treatment induced substantial increases in intratumoral T cells and a decrease in immunosuppressive regulatory T cells. This immunity was critically dependent on CD8^+^ T cells and less dependent on CD4^+^ T cells. Our data provide significant support for the continued development of VC2 as an OVT for the treatment of human and animal cancers.

**IMPORTANCE** Current oncolytic virotherapies possess limited response rates. However, when certain patient selection criteria are used, oncolytic virotherapy response rates have been shown to increase. This, in addition to the increased response rates of oncolytic virotherapy in combination with other immunotherapies, suggests that oncolytic viruses possess significant therapeutic potential for the treatment of cancer. As such, it is important to continue to develop novel oncolytic viruses as well as support basic research into their mechanisms of efficacy. Our data demonstrate significant clinical potential for VC2, a novel type 1 oncolytic herpes simplex virus. Additionally, due to the high rates of survival and the dependence on CD8^+^ T cells for efficacy, our model will enable study of the immunological correlates of protection for VC2 oncolytic virotherapy and oncolytic virotherapy in general. Understanding the mechanisms of efficacious oncolytic virotherapy will inform the rational design of improved oncolytic virotherapies.

## Abstract (precis)

Current oncolytic virotherapies possess limited response rates. However, when certain patient selection criteria are used, oncolytic virotherapy response rates have been shown to increase.

## INTRODUCTION

Immunotherapy, the targeted alteration of immunological parameters to achieve a therapeutic outcome, has revolutionized the treatment of cancer. Immunotherapeutic approaches to the treatment of cancer include vaccination, oncolytic virotherapy (OVT), chimeric antigen receptor T cells (CAR-T), and checkpoint inhibitors. While these approaches have met with great success, there are still many drawbacks to current immunotherapeutic modalities, which include limited response rates and the serious side effects seen with CAR-T and checkpoint inhibitors (1–3).

The first FDA-approved oncolytic virus, T-Vec, demonstrates the therapeutic potential and safety of OVT. However, thus far T-Vec has achieved limited response rates (4). More recent studies suggest that T-Vec monotherapy can be improved when specific patient selection criteria are used (5). Additionally, the ability of oncolytic viruses to promote an infiltration of antitumor T cells has been shown to improve the utility of checkpoint inhibitors as a combination therapy (6–8). These examples demonstrate the promise of OVT and support a need for improved oncolytic viruses that are capable of facilitating robust and long-lasting antitumor immune responses.

Establishment or reestablishment of the cancer-immunity cycle is the basis of immunotherapy. The cancer-immunity cycle begins with liberation of tumor-associated antigens (TAAs) via spontaneous immunogenic cell death, radiation, chemotherapy, and/or OVT, among others (9). Antigen-presenting cells traffic from tumors to draining lymph nodes to prime and activate tumor-specific T cells. Tumor-specific T cells then traffic to and infiltrate the tumor, whereby recognition of TAAs on cancer cells can lead to eradication of tumors. It is widely held that the development of tumor-infiltrating lymphocytes (TILs), primarily CD8^+^ T cells, is critical for immune control of tumors (10–12).

Historically OVT was proposed largely to shrink tumors via replication and spread through transformed cells. OVT is now, however, understood to be a bona fide immunotherapy, whereby lysis of tumor cells liberates TAAs in the context of immunogenic viral infection, leading to the development of antitumor immunity (13). In support of this idea, recent immunogenetic analyses of more than 10,000 tumors defining tumor-extrinsic signatures capable of predicting outcomes found that gene signatures associated with host antiviral responses were correlated with increased survival (14). The tumor microenvironment (TME) is made up of a number of cellular and subcellular constituents and is now understood to be largely responsible for mediating the immunosuppression that allows tumors to grow and, in the case of cancer, to spread (15). Mechanistically, it is increasingly understood that the context of viral infection can disrupt immunosuppression in the TME (16–18). This reversal of immunosuppression in the TME can result in the infiltration and proliferation of CD8^+^ T cells that suppress tumor growth (19, 20).

A number of virus species are being developed as oncolytic virotherapies, including Newcastle disease virus (NDV), measles virus, and adenovirus, among others (21, 22). The only FDA-approved OVT, T-Vec, is a type 1 herpes simplex virus (HSV-1) (23). Herpes simplex viruses possess many qualities that inform their usage as an OVT, including their relative safety, large coding capacity, highly tractable genetics system, wealth of molecular virology data, and the ability to reinfect hosts multiple times (24–27). The natural history of HSV-1 is characterized by the establishment of a primary infection in the epithelium, with subsequent infection of innervating axonal termini (28). The virus then travels to the nucleus of neurons, where it will establish and maintain latent infection for the life of the host. Periodically, the virus will reactivate from latency and cause clinical symptoms, most often seen as lesions in and around the mouth. Additionally, in a minority of cases, reactivation can cause significant morbidity and even mortality, as seen with herpes encephalitis or keratitis (29, 30).

Our laboratory has generated an HSV-1 mutant unable to enter axons via axonal termini (31–33). The inability to enter neurons abolishes the establishment and maintenance of a latent infection and thus greatly improves its safety profile. This virus, VC2, possesses a deletion of 38 amino acids in the N terminus of the viral envelope glycoprotein K (gK), which is highly conserved among all alphaherpesviruses (34). We have shown that this mutation in the N terminus of gK precludes entry by fusion and forces the virus to enter cells via endocytosis (35). Additionally, VC2 possesses a deletion of amino acids 4 to 22 of a second envelope protein, UL20. VC2 is currently being developed as a vaccine against both HSV-1 and HSV-2, and we have shown that this virus is safe and protective in mouse, guinea pig, and macaque models (36–41).

In this article, we show that treatment of melanoma tumors in a modified B16F10 mouse melanoma model reduced tumor size and increased survival over control treatment. Importantly, VC2 OVT promotes a long-lasting systemic antitumor immunity that is dependent on the development of an antitumor CD8^+^ T-cell response. These data support the continued development of VC2 as a safe, effective OVT.

## RESULTS

### VC2 oncolytic virotherapy reduces tumor growth and enhances survival in an immunocompetent murine melanoma model.

**FIG 1:**
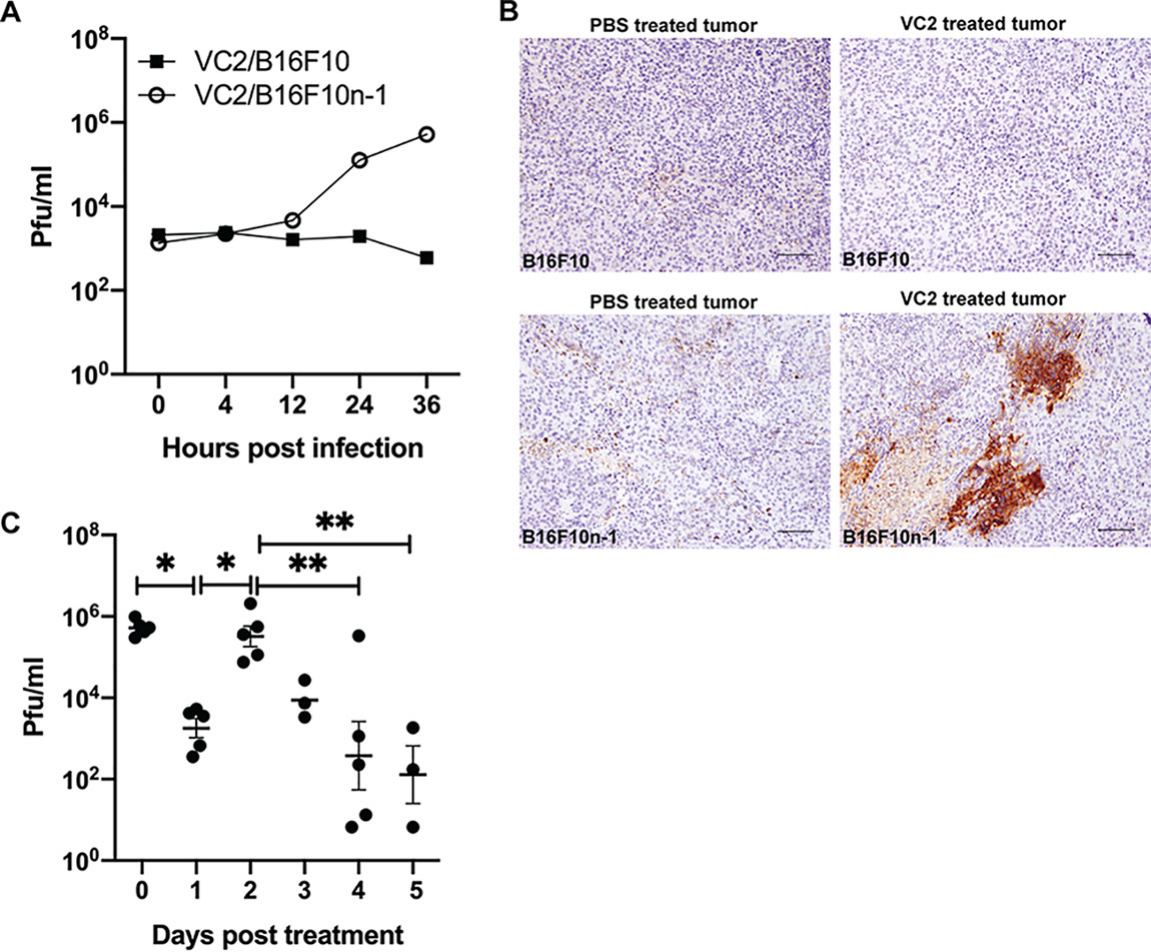
VC2 replicates in B16F10 tumors expressing nectin-1. (A) *In vitro* VC2 infection (multiplicity of infection [MOI] of 0.01) of either B16F10 cells or B16F10n-1 (cells transduced with nectin-1). Supernatants and cell pellets, were harvested at indicated times postinfection, and plaque assays were performed to determine viral titers. (B) Seventy-two hours after treatment of engrafted tumors with either PBS or VC2, mice were sacrificed, and B16F10 tumors or B16F10n-1 tumors were excised, processed for immunohistochemistry, and stained with anti-HSV antibody. Representative images from three mice per group are shown. (C) At indicated days posttreatment with VC2, mice were sacrificed, and B16F10n-1 tumors were removed and sonicated. Virus was quantified by plaque assay. *n* = 5 mice per group. *, *P* < 0.05; **, *P* < 0.005.

**FIG 2:**
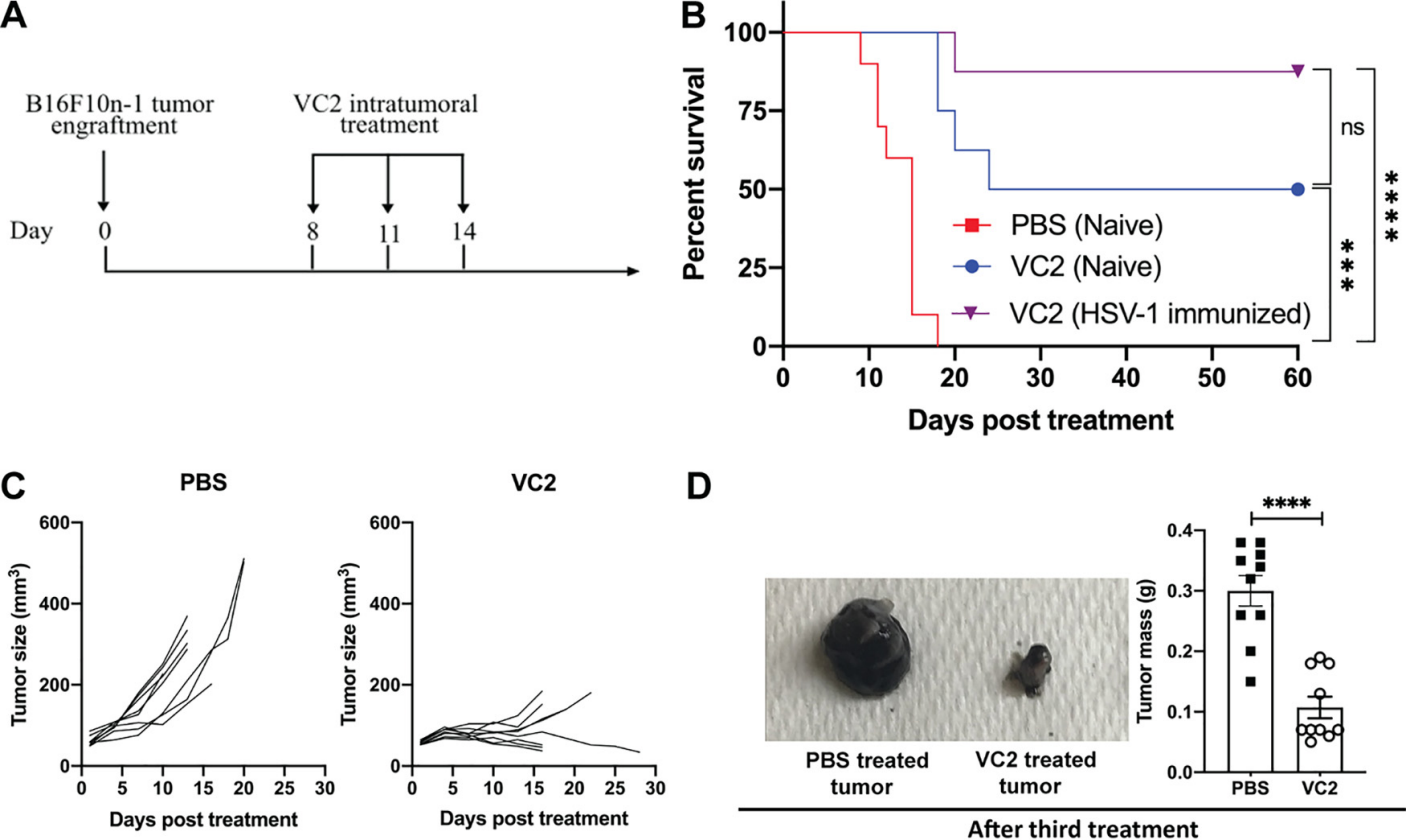
VC2 reduces tumor growth rates and enhances survival in an immunocompetent melanoma model. (A) Timeline of treatment regimen. Engrafted B16F10n-1 tumors were treated, beginning when tumors reached 50 to 100 mm^3^, every 3 days for a total of three treatments. Mice were sacrificed when tumors reached greater than 1,000 mm^3^ or the mice became excessively moribund. (B) Kaplan-Meier survival curves. (C) Over the course of treatment with either VC2 or PBS, tumor volumes were measured and growth rates determined until the first death. (D) Image of PBS- or VC2-treated tumors removed one day after the third treatment and quantification of tumor mass. *n* = 8 to 10 mice per group. ***, *P* < 0.0005; ****, *P* < 0.00005.

To study the efficacy of VC2 in a B16F10 syngeneic mouse model of melanoma, we developed a modified B16F10 melanoma model. B16F10 cells lack nectin-1, the receptor for HSV-1, and are thus refractory to infection by HSV (42). To overcome this limitation, B16F10 cells were transduced with lentivirus to express nectin-1. Transduced cells (B16F10n-1) were selected for resistance to hygromycin B and for their ability to support HSV-1 replication. *In vitro* growth analysis of VC2 in B16F10 cells revealed no growth, while VC2 grew to titers of 10^6^ PFU in B16F10n-1 cells (Fig. 1A). To determine the ability of VC2 to replicate in engrafted tumors, B16F10n-1 cells were engrafted intradermally (caudal to the ear pinna), and at approximately 8 days postengraftment, when tumors reached a volume of 50 to 100 mm^3^, either phosphate-buffered saline (PBS) or 1 × 10^6^ PFU VC2 was introduced intratumorally in a volume of 100 μl. Three days posttreatment, tumors were removed and immunohistochemistry (IHC) was performed to detect the presence of virus. Virus was readily detected in tumors treated with VC2 compared to tumors treated with PBS (Fig. 1B). To develop a protocol for treatment of engrafted tumors, we determined the ability of engrafted tumors to support replication by HSV-1. To determine the replication status of VC2 in tumors after treatment, we quantified virus in once-treated tumors that were removed at different times posttreatment (Fig. 1C). We were able to detect input virus at day 0, and we noted a 3-log drop in virus titer 1 day after treatment. Day 2 posttreatment, titers reached 10^6^ PFU, and after day 2, there was a steady decrease in viral titers out to day 5 posttreatment (Fig. 1C). Using these data to inform the treatment protocol, we decided to administer VC2 intratumorally every third day (three total treatments) to keep virus titers as high as possible (Fig. 2A). Importantly, using this treatment protocol we were unable to detect any virus in the lung, spleen, liver, or nervous system of treated mice after treatment 3 (data not shown).

To determine the efficacy of VC2 as an OVT, we performed a survival analysis. B16F10n-1 cells were engrafted intradermally, caudal to the ear pinna, and treated every third day for three total treatments (Fig. 2A). When tumors reached greater than 1,000 mm^3^ or mice were excessively moribund, they were sacrificed. Mice for which tumors were treated with PBS all required sacrifice before 20 days posttreatment (Fig. 2B). In contrast, 50% of mice for which tumors were treated with VC2 survived (Fig. 2B). Overall, tumors treated with PBS exhibited a high rate of growth prior to sacrifice (Fig. 2C and D). Some VC2-treated tumors shrank rapidly before becoming undetectable, whereas others grew steadily, albeit much more slowly than those that were PBS treated (Fig. 2C and D). Due to the significant global burden of HSV-1 infection (43), it is important to examine the efficacy of HSV-1-based OVT in seropositive animals. To determine the effect of HSV-1 seropositivity on VC2 efficacy in our model, mice were exposed intramuscularly to parental F strain virus 30 days prior to engraftment and subsequent treatment of tumors with VC2. We observed no significant differences in the efficacy of VC2 OVT between naïve mice and mice that had been previously exposed to HSV-1 (Fig. 2B). This is consistent with a number of reports that found no differences in efficacy of HSV-1-derived OVT in mice that had been preexposed to HSV-1 (44, 45).

### VC2 treatment affects intratumoral T-cell populations.

**FIG 3:**
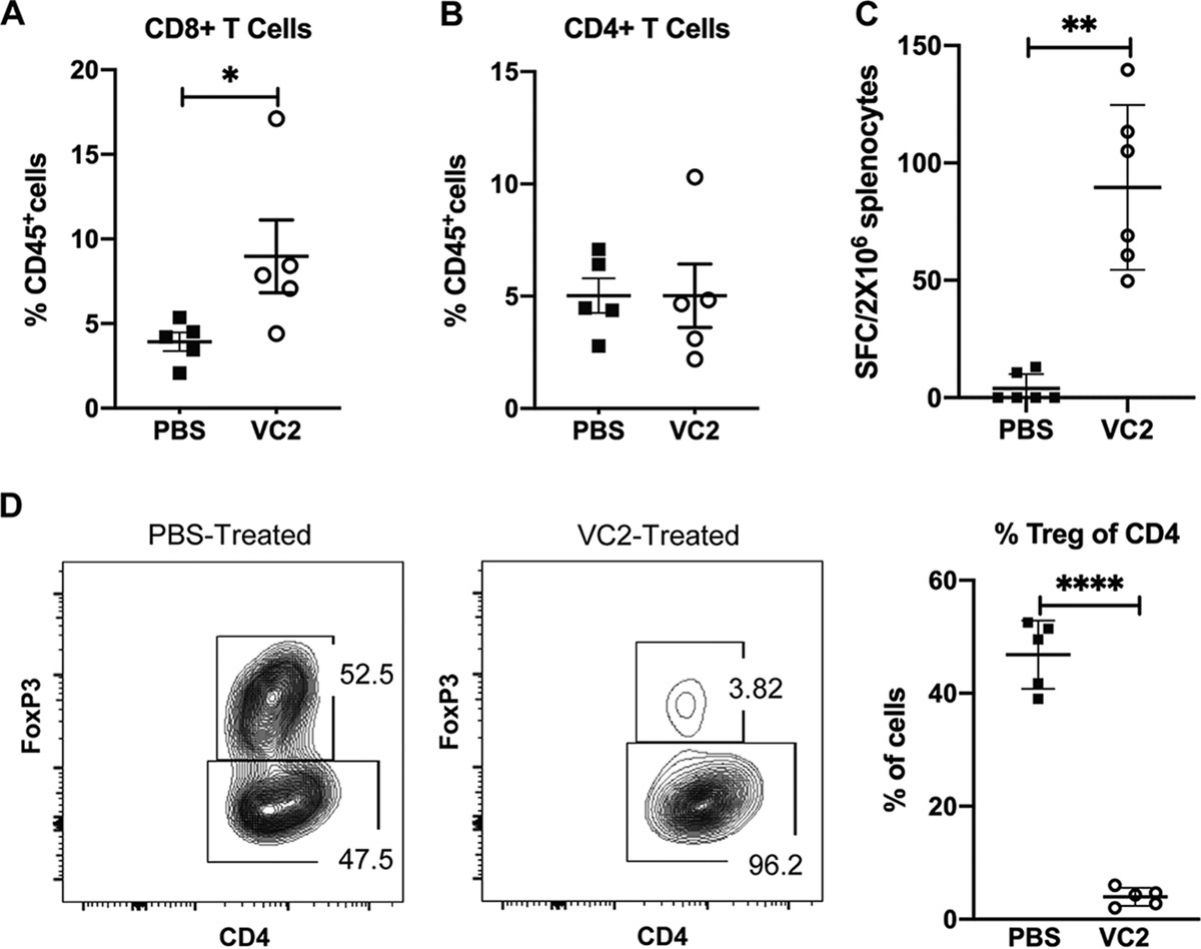
VC2 treatment promotes B16F10n-1-specific T cells and reduces T regulatory cells (Tregs) in the tumor. (A and B) Flow cytometry quantification of intratumoral CD8^+^ and CD4^+^ T cells 1 day after the third treatment. (C) Mixed-lymphocyte assay. Mice were engrafted with B16F10n-1 cells and treated with either VC2 or PBS as in Fig. 2. One day after the third treatment, spleens were removed from mice. Splenocytes (2 × 10^6^) were isolated and cultured in a 20:1 ratio with mitomycin C-treated B16F10n-1 cells, and IFN-γ-producing cells were quantified by ELISPOT assay. (D) Flow cytometry quantification of intratumoral Tregs 1 day after the third treatment. Relative percentages of Tregs in the tumor as a percentage of CD4^+^ cells are shown. *n* = 5 mice per group. *, *P* < 0.05; **, *P* < 0.005; ****, *P* < 0.00005.

To determine the effect of VC2 OVT on T-cell populations in B16F10n-1 tumors, flow cytometry experiments were performed on tumors removed 1 day after the third treatment with either the PBS control or VC2. Tumors were removed, and single-cell suspensions were stained with antibodies against CD45, CD3, CD4, CD8, and FoxP3. As a percentage of CD45^+^ cells, CD8^+^ T cells were found to significantly increase in tumors treated with VC2 compared to tumors treated with PBS (Fig. 3A). We did not detect any differences in the CD4^+^ populations in tumors after treatment with VC2 compared to treatment with PBS (Fig. 3B).

To determine whether VC2 treatment of tumors was generating specific antitumor T-cell responses. We performed a gamma interferon (IFN-γ) enzyme-linked immunosorbent spot (ELISPOT) assay using splenocytes extracted from mice 1 day after the third treatment with PBS or VC2. Splenocytes were mixed with mitomycin C-treated B16F10n-1 cells. With splenocytes extracted from mice that had tumors treated with PBS, we detected few spot-forming colonies (Fig. 3C). In contrast, we detected more than 100 spot-forming colonies per 2 × 10^6^ splenocytes from many of the mice that had tumors treated with VC2 (Fig. 3C).

Regulatory T cells (Tregs) suppress antitumor responses, and an abundance of these cells is associated with a poor outcome for cancer patients (46). These cells make a significant contribution to immunosuppression in the TME and are a target for developing immunotherapeutics (47). We used flow cytometry to determine whether VC2 treatment affected Treg numbers in B16F10n-1 tumors. We found that after treatment of tumors with VC2, there was a profound loss of Tregs compared to tumors treated with PBS (Fig. 3D).

**FIG 4:**
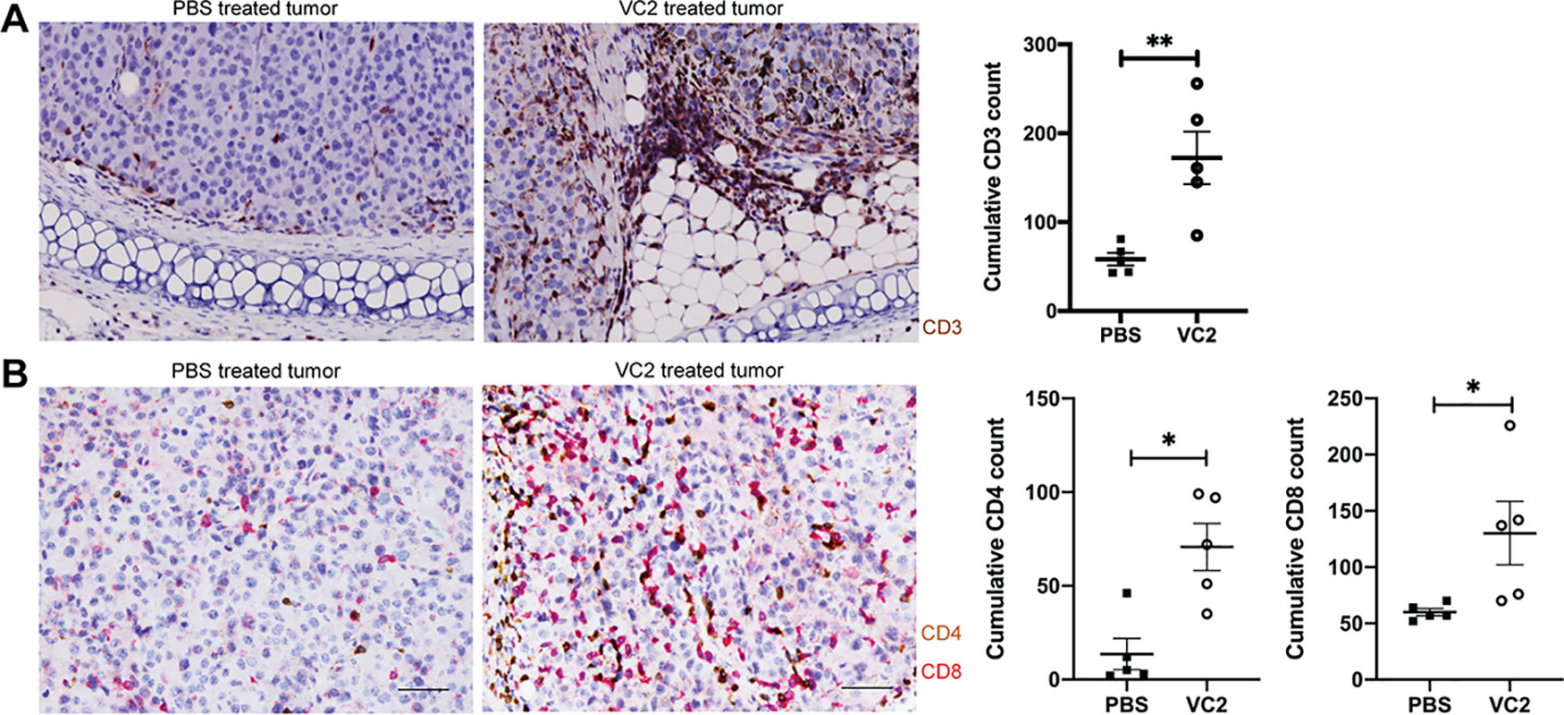
VC2 treatment promotes infiltration of T cells into tumors. (A) PBS- or VC2-treated B16F10n-1 tumors were removed from mice 1 day after the third treatment and processed for immunohistochemistry. Tissues were stained with anti-mouse CD3 (brown) antibody and a counterstain. (B) CD4^+^ (brown) and CD8^+^ (red) double staining of the same tumors. Scale bar, 50 μm. Immunostaining was scored based on the cumulative number of positive cells in five high-magnification (40×) microscopic fields. Representative images from 5 mice per group are shown. *, *P* < 0.05; **, *P* < 0.005.

We wished to determine whether T cells in tumors after treatment with either PBS or VC2 were infiltrating tumors or remaining in the periphery. To achieve this, we performed immunohistochemistry experiments. One day after the third treatment, mice were sacrificed, and tumors were excised and processed for immunohistochemistry. Tissue sections were stained with anti-CD3 antibody to identify T cells in tumors after treatment with either VC2 or the PBS control. It was readily apparent that VC2 treatment of tumors resulted in a greater number of tumor-infiltrating T cells compared to PBS-treated tumors (Fig. 4A).

To determine whether the CD3^+^ cells were CD4^+^ or CD8^+^, we performed double staining on sections from the tumors we had used for CD3 staining described above. Staining of tissue revealed an increase in cumulative numbers of both CD4^+^ and CD8^+^ T cells in tumors treated with VC2 (Fig. 4B). Furthermore, there appeared to be approximately twice as many CD8^+^ T cells in tumors treated with VC2.

### T cells are essential for VC2 efficacy.

**FIG 5:**
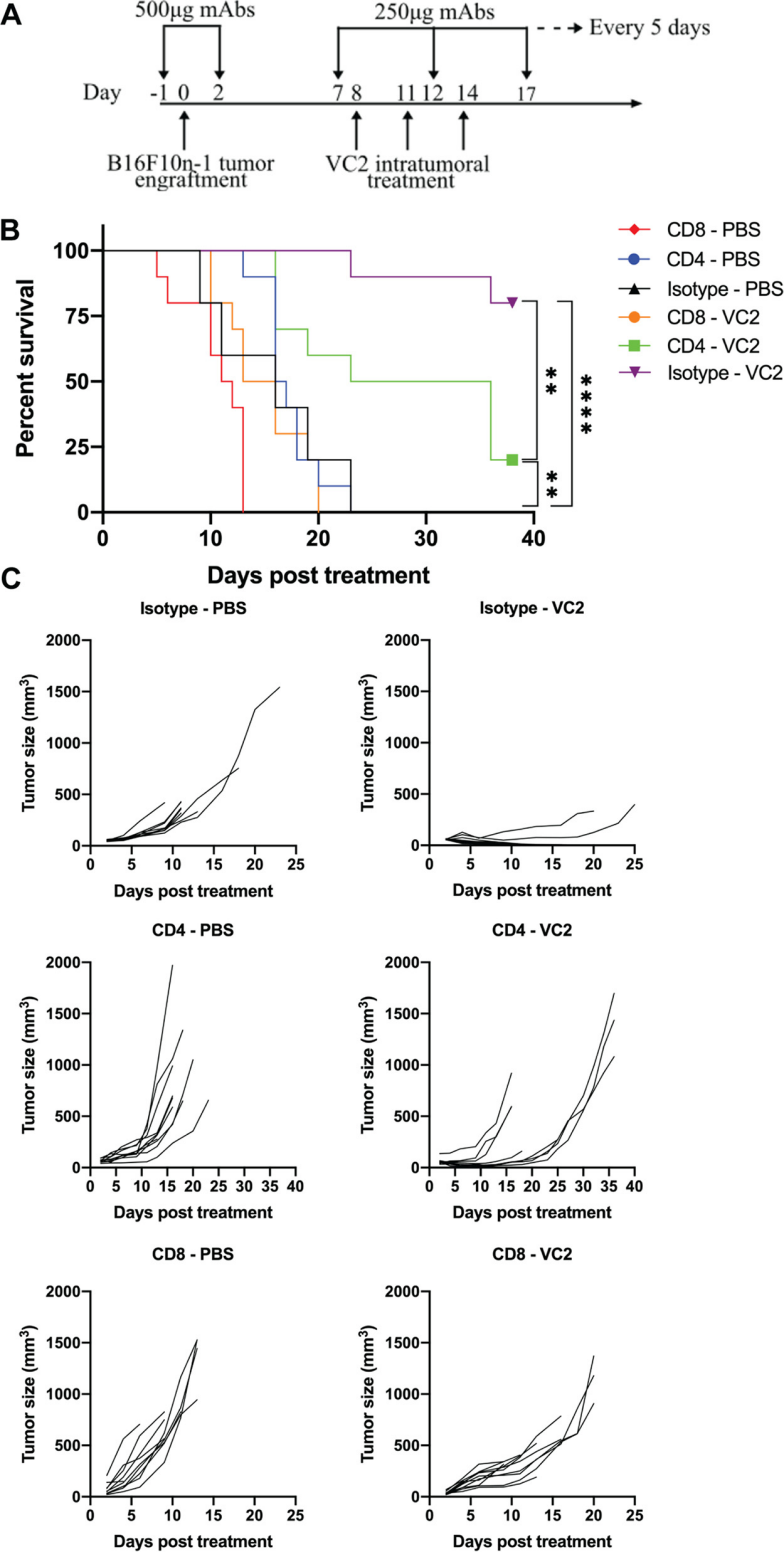
CD8^+^ T cells are required for VC2 efficacy. (A) Treatment and depletion regimen. Mice were intraperitoneally administered 500 μg of either isotype control or CD4- or CD8-depleting antibodies 1 day before and 2 days after B16F10n-1 tumor engraftment and continued intraperitoneal treatments with 250 μg of the appropriate MAbs every 5 days. B16F10n-1 tumor-bearing depleted and control mice were treated with either VC2 or PBS as in Fig. 2. (B) Kaplan-Meier survival curve and (C) tumor growth rates of mice from the survival study. *n* = 9 to 10 mice per group. **, *P* < 0.005; ****, *P* < 0.00005.

T-cell responses have been shown to be critical effectors of OVT efficacy. Our flow cytometry profiling of T-cell populations over the course of OVT with VC2 suggests a possible role for T cells in VC2 efficacy. To test the contribution of T cells to VC2 efficacy, we performed antibody depletion assays. Mice were depleted of either CD4^+^ or CD8^+^ T cells or treated with isotype antibody 1 day prior to engraftment, at 2 days postengraftment, and every 5 days afterwards (Fig. 5A). One hundred percent of mice depleted of CD8^+^ T cells and treated with VC2 required sacrifice within 25 days (Fig. 5B). CD4^+^ T-cell depletion appeared to also be important for VC2 efficacy, as 80% of mice depleted of CD4^+^ T cells and treated with VC2 required sacrifice, albeit at much later time points than mice depleted of CD8^+^ T cells (Fig. 5B). Differences in tumor growth rates were consistent with survival data (Fig. 5C).

### VC2 induces long-lasting, systemic antitumor immunity.

**FIG 6:**
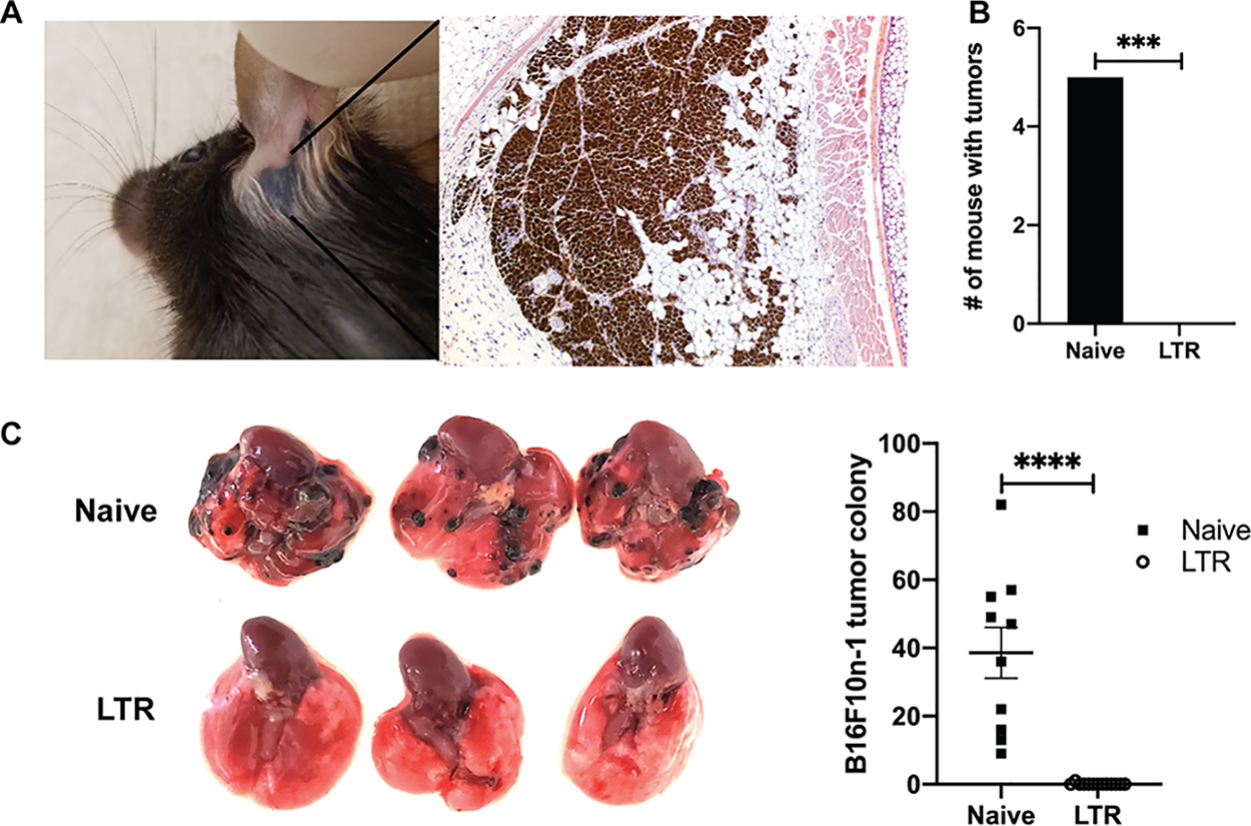
VC2 treatment promotes durable, systemic antitumor responses. (A) H&E staining of tissue removed from mice that survived initial engraftment after treatment with VC2 (long-term responders [LTR]). (B) Number of B16F10n-1 tumor-bearing mice after reengraftment. *n* = 5 for naïve and 10 for LTR mice. (C) B16F10n-1 cells were introduced intravenously into LTR (*n* = 13) or naïve (*n* = 10) mice. Representative images of lungs from naïve and LTR mice 3 weeks postinoculation and quantification of colonization are shown. ***, *P* < 0.0005; ****, *P* < 0.00005.

VC2-treated mice that survived the initial engraftment were called long-term responders (LTRs). We noted that LTR mice possessed a black spot where the tumor had been engrafted. There was no palpable tumor present. To more closely examine the nature of the black spots, we performed a biopsy and hematoxylin and eosin (H&E) staining (Fig. 6A). A pathologist’s examination of slides generated from biopsied tissue revealed no tumor cells but rather a large number of macrophages that were filled with melanin, likely the remains of the engrafted tumor.

To determine whether treatment with VC2 endowed mice with long-term immunity to the engraftment of B16F10n-1 cells, these cells were reengrafted intradermally contralateral to the initial engraftment in LTR mice or naïve mice. We successfully engrafted all of the naïve mice, whereas 0/10 engraftments produced tumors in LTR mice (Fig. 6B).

Intradermal engraftment of B16F10 cells does not readily lead to metastasis in this mouse model. However, it is possible to introduce these cells intravenously, after which B16F10 cells are known to colonize mouse lungs (48). To further examine the extent of antitumor immunity of LTR mice, we intravenously introduced B16F10n-1 cells into either naïve mice or LTR mice. After 3 weeks, tumors were readily detected in the lungs of naïve mice, whereas only one tumor was detected in any of the successfully treated mice (Fig. 6C).

### VC2 OVT decreases growth rates of distant, untreated tumors.

**FIG 7:**
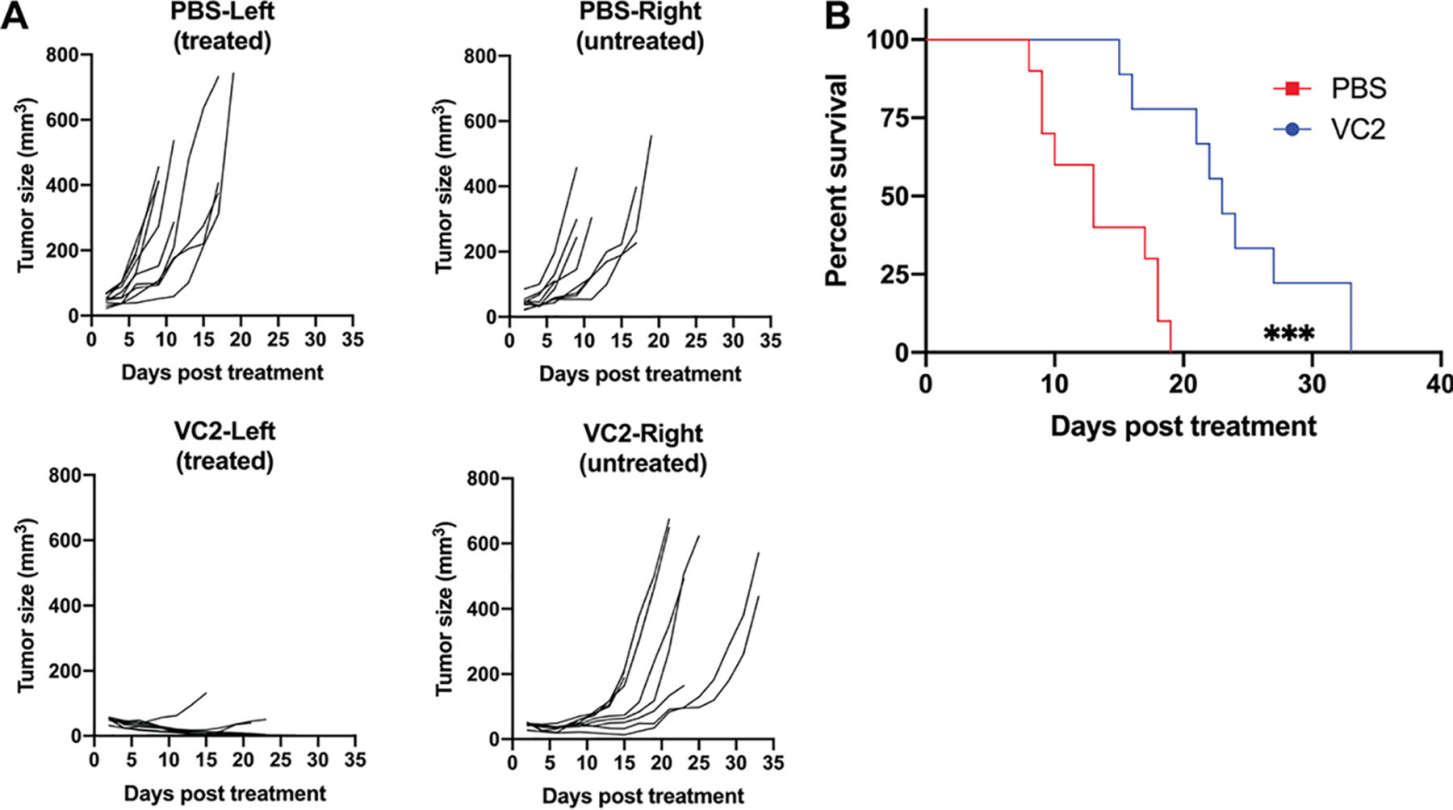
VC2 treatment delays the growth rate of untreated distal tumor and prolongs survival. Both the left and right sides (caudal to the ear pinnae) of mice were engrafted intradermally with 5 × 10^5^ B16F10n-1 cells. Tumors on the left were treated with either PBS or VC2 every 3 days for a total of three treatments. Mice were sacrificed when either tumor reached greater than 1,000 mm^3^ or mice became excessively moribund. (A) Individual tumor growth rates and (B) Kaplan-Meier survival curve. Median survival times with PBS (13 days) and VC2 (23 days) are shown. *n* = 9 to 10 mice per group. ***, *P* < 0.0005.

The ability to induce antitumor immunity in untreated tumors that are distant from treated tumors is referred to as the “abscopal” effect (49). To address the ability of VC2 treatment to induce antitumor immunity in untreated tumors, we engrafted two tumors intradermally, caudal to each ear of each mouse. Treatment with either PBS or VC2 was initiated on the left tumor when these tumors reached approximately 50 to 100 mm^3^. Tumors treated with PBS exhibited similar growth rates to contralateral, untreated tumors. In these mice, both tumors continued to grow, requiring sacrifice of mice within 20 days of initiation of treatment (Fig. 7A). This was in contrast to mice in which the left tumor was treated with VC2. In these mice, treated tumor growth rates were similar to those in previous experiments where only one tumor was engrafted, while the untreated tumors in these mice exhibited decreased growth rates (Fig. 7A). While mice that had tumors treated with VC2 all eventually required sacrifice due to the growth of untreated tumors, these mice exhibited a significant increase of 10 days in median survival time compared to mice that had tumors treated with PBS (Fig. 7B).

## DISCUSSION

Beyond safety, an important characteristic of an effective oncolytic virus is the ability to induce strong, durable, and systemic antitumor cell-mediated immune responses. Our principal findings are that in an aggressive B16F10-derived melanoma model, VC2 OVT slows tumor growth rates, facilitates greater than 50% survival, and promotes long-term, systemic antitumor immunity. Furthermore, our depletion and functional studies strongly suggest that the efficacy of VC2 OVT is due to its ability to induce antitumor CD8^+^ T cells.

The induction of antitumor CD8^+^ T cells is a primary goal of immunotherapies, including OVTs. Many herpesvirus-derived OVTs have demonstrated significant antitumor CD8^+^ T-cell responses as a mechanism of their efficacy (26, 44, 50, 51). A recent study of the efficacy of T-Vec in combination with checkpoint inhibitors found that the infiltration of CD8^+^ T cells into tumors was highly correlated with individuals who responded to combination therapy (52). While not explicitly tested in our experiments, the immunity to intravenous B16F10 challenge of LTR mice suggests the development of significant CD8^+^ T-cell memory responses.

Evidence of the importance of CD4^+^ T cells in our model is suggested by our depletion studies, in which depletion of CD4^+^ T cells resulted in decreased efficacy of VC2 OVT. We were able to detect statistically significant increases in intratumoral CD4^+^ T cells via IHC. However, we were unable to support this via flow cytometry. We suggest that IHC examination is better suited to discriminate intratumoral CD4^+^ T cells from CD4^+^ T cells that are restricted to the tumor periphery. It is also important to note that CD4^+^ T cells have been demonstrated to facilitate optimal antitumor CD8^+^ T-cell responses and that this role for CD4^+^ T cells is not necessarily restricted to intratumoral CD4^+^ T cells (53, 54). As such, our data are consistent with a role for CD4^+^ T cells in optimization of an antitumor immune response during VC2 OVT in our model.

What distinguishes VC2 from other herpesvirus-derived OVTs, beyond its efficaciousness, is that it does not express transgenes such as those coding for interleukin-12 (IL-12) or granulocyte macrophage colony-stimulating factor (GM-CSF). These transgenes were initially proposed to promote the infiltration of immunogenic cells, such as dendritic cells, which may promote development of antitumor immunity. This is a significant difference between VC2 and other herpesvirus-derived OVTs, including T-Vec. Early approaches to the generation of safe herpesvirus-derived OVT included deletion or mutation of genes that were found to attenuate virus replication in normal tissues but did not affect virus replication and spread through transformed cells (45, 55). More recent approaches to restriction of OVT to transformed cells include the insertion of microRNA (miRNA) target sites in critical HSV genes, use of specific promoters to express critical HSV genes, and receptor retargeting (26, 56–58). However, all of these approaches possess limitations, including the possibility of reversion of attenuation, in addition to compromised replicative potential and thus efficacy. VC2 replicates to similar viral titers to its parental virus, HSV-1(F), with the exception that it cannot enter neuronal axons and establish latency in ganglionic neurons. More recently, it has been proposed that more fully replication-competent oncolytic herpesviruses, such as VC2, may exhibit greater efficacy as OVTs (26, 44, 59). Our results are in agreement with this idea, and we speculate that the ability of VC2 to replicate well in nonneuronal cells contributes to its efficacy and what may be its enhanced immunogenicity in comparison to other herpesvirus-derived OVTs.

For virus-based therapies such as OVTs, preexisting immunity is a concern as preexisting immunity may result in a decrease in efficacy. This is due to the effect antiviral immunity has on the ability of OVT to induce an antitumor immune response prior to being eliminated by an adaptive immune response. This is of particular concern with HSV-1-based therapeutic and prevention strategies due to the high prevalence of HSV-1 exposure in the human population (43). We demonstrate here that preexposure of mice to HSV-1 prior to engraftment and subsequent treatment has no effect on VC2 OVT in our model. This is consistent with a number of groups reporting similar results (44, 45). It is worth noting that other groups have successfully leveraged preexisting antiviral immunity to enhance therapeutic outcome (60–62). It has been suggested that this may be particularly true for herpesviruses, which as part of their replicative strategy periodically reactivate and spread in the presence of significant adaptive immune responses (62). As such, rather than being a hindrance to herpesvirus-based OVTs, preexisting immunity may enhance the efficacy of such therapies.

In addition to the ability of VC2 to replicate well in nonneuronal cells, it may be that the alteration of initial host-pathogen interactions due to the mutations in HSV-1 envelope proteins gK and UL20 in VC2 lead to differential host responses that are more immunogenic than during wild-type virus infection. VC2 enters into epithelial and other cell types exclusively via endocytosis, which may enhance innate immune responses, leading to enhanced cellular responses. This is supported by our finding that intramuscular immunization with VC2 fully protected against ocular challenge of mice with the human clinical strain HSV-1(McKrae), while immunization with its parental virus, HSV-1(F), conferred only partial protection (41). The development of vectors for the induction of strong T-cell-mediated immune responses are needed for use in vaccination against infectious disease and cancer (63). The potential increase in immunogenicity may facilitate the translational potential of VC2 to serve as a vaccine vector and OVT.

We show here that VC2 treatment significantly reduced the number of Tregs in tumors. This strongly suggests that VC2 treatment reverses the immunosuppression in the TME as a mechanism of its efficacy. VC2-treated mice that survived the intradermal engraftment responded very quickly (within days) to treatment. This also suggests that rather than the development of a *de novo* antitumor T-cell response, the efficacy of VC2 treatment in our model is due to a reversal of immunosuppression, leading to rapid expansion of antitumor T cells in VC2-treated tumors. In this respect, it is important to note that our model can be used to study the cellular, molecular, and immunological mechanisms of oncolytic virus efficacy. Specifically, it will be important to determine how VC2 affects the TME as a component of efficacy. A major focus of our work moving forward will be to examine the cellular and subcellular constituents of the TME during VC2 OVT. These studies will inform the rational design of improved oncolytic viruses for the treatment of human cancers.

## MATERIALS AND METHODS

### Animals.

Female C57BL/6J mice were purchased at ages of 4 to 5 weeks from the Jackson Laboratory (Bar Harbor, ME). All animals were fed with a regular rodent diet and housed under standard conditions, with no more than 5 per cage under specific-pathogen-free conditions, in a temperature-controlled room with a 12-h light/12-h dark cycle. All procedures involving animals and their handling were conducted in accordance with the National Institutes of Health guidelines and were approved by the Louisiana State University Institutional Animal Care and Use Committee (IACUC reference no. 20-002).

### Construction of the VC2 virus.

The VC2 virus was constructed using the two-step double-Red recombination method implemented on the HSV-1(F) genome cloned into a bacterial artificial chromosome (BAC), as previously described (64). The VC2 virus harbors mutations in two of its viral envelope proteins: glycoprotein K (gK) and UL20 (36). VC2 virus was grown and its titer determinted in Vero cells (ATCC), as described previously (65).

### Cell culture.

B16F10 murine melanoma cells were obtained from American Type Culture Collection (Manassas, VA). B16F10 cells were grown in Dulbecco's modified Eagle's medium (DMEM) supplemented with 10% filtered, heat-inactivated fetal bovine serum (FBS; Gibco-BRL, Grand Island, NY) and 100 μg/ml Primocin (InvivoGen, San Diego, CA). African green monkey kidney (Vero) cells were cultured in DMEM containing 10% FBS and 100 μg/ml Primocin.

### Human nectin-1 stable transduction.

The B16F10 cell line was transduced with a third-generation lentiviral particle-packaged vector produced by Vector Builder (Cyagen) to stably express human nectin-1. The nectin-1 was tagged with the red fluorescent protein mCherry and was expressed under the control of a cytomegalovirus (CMV) promoter. Hygromycin selection was used to enrich stable B16F10 cells expressing nectin-1.

### Tumor engraftment and treatment regimens.

Mice were anesthetized with 2 to 3% isoflurane, and B16F10n-1 cells were engrafted orthotopically in the dermis of the dorsal left dorsal pinna, as previously reported (66). Mice were engrafted with 5 × 10^5^ B16F10n-1 cells in 100 μl PBS. When tumors reached 50 to 100 mm^3^, mice were intratumorally injected with either PBS or 1 × 10^6^ PFU VC2 in volumes of 100 μl every 3 days, unless otherwise indicated. Tumors were measured approximately every 2 to 3 days with a digital caliper, and tumor volumes were calculated by using the formula 1/2(length × width^2^). Tumor-bearing mice were euthanized when tumors reached greater than 1,000 mm^3^ or when mice were excessively moribund. For the abscopal experiments, 5 × 10^5^ B16F10n-1 cells were engrafted in the dermis, caudal to both the left and right dorsal pinnae of each mouse. Approximately 8 days postengraftment, when tumors reached approximately 50 to 100 mm^3^, the tumor on the left dorsal pinna was directly injected with 1 × 10^6^ PFU VC2 or PBS. Tumor volume was monitored every 2 days, and animals were euthanized when tumors reached greater than 1,000 mm^3^ or when mice were excessively moribund.

### ELISPOT assays.

One day after the third treatment, mice were sacrificed and spleens were removed. Splenocytes (2 × 10^6^) were isolated and cultured overnight with mitomycin C-treated B16F10n-1 cells (ratio of 20:1). IFN-γ-producing splenocytes were quantified according to the manufacturer’s instructions using an Immunospot (Shaker Heights, OH) murine IFN-γ single-color ELISPOT assay.

### Viral titration assay.

B16F10n-1 tumor-bearing mice were injected intratumorally with a single dose of 1 × 10^6^ PFU VC2. B16F10n-1 tumors were harvested at 0, 24, 36, 48, 60, and 72 h posttreatment. Tumors were weighed, and DMEM was added according to tumor weight (1 ml DMEM per 0.1 g tumor) and stored at −80°C until further use. Each tumor sample was subjected to sonication using a Branson 250 Sonifier (equipped with a microtip), set at an output control of ∼3.5 and a duty cycle of 45, with an intermittent 10 s of cooling on ice. Supernatants from each tumor sample were subjected to 1:10 serial dilutions, which were inoculated on a 95% confluent 12-well plate of Vero cells and rocked for 1 h. Following rocking, infection medium was removed and replaced with methylcellulose overlay medium (DMEM containing 0.5% methylcellulose and 5% FBS). Each plate was incubated at 37°C in an atmosphere of 5% CO_2_ for 3 to 4 days and fixed with 3.5% formalin overnight at room temperature. Cells were washed, and then incubated with anti-HSV-1 antibody (Agilent, Santa Clara, CA) for 1 h, followed by the addition of polyclonal goat anti-rabbit Igs conjugated to horseradish peroxidase (HRP) (Agilent, Santa Clara, CA) for 30 min at room temperature. Viral plaques were visualized by application of a NovaRED peroxidase (HRP) substrate (Vector, Burlingame, CA) and counted under a microscope.

### Lung colonization assay.

LTR or naïve mice were injected intravenously with 5 × 10^5^ B16F10n-1 cells in 100 μl PBS. After 3 weeks, mice were sacrificed, lungs were removed, and the tumor colonies on the lung surface were counted.

### Dual immunohistochemistry.

For dual immunohistochemistry (IHC), sections of formalin-fixed, paraffin-embedded (FFPE) tissues (4 μm) were mounted on positively charged Superfrost Plus slides (Fisher Scientific, Pittsburgh, PA) and processed using the automated BOND-MAX system (Leica Biosystems, Buffalo Grove, IL) as previously described (67, 68). Dual immunostaining for CD4 and CD8 was performed sequentially using the Bond Polymer Refine Detection and Bond Polymer Refine Red Detection kits, respectively. Following automated deparaffinization, tissue sections were subjected to automated heat-induced epitope retrieval (HIER) using a ready-to-use EDTA-based solution (pH 9.0; Leica Biosystems) for 20 min at 100°C. Subsequently, endogenous peroxidase was quenched by incubation with 3% hydrogen peroxide (5 min), followed by incubation with a recombinant rabbit anti-mouse CD4 monoclonal antibody (MAb) (clone EPR19514; Abcam, Cambridge, United Kingdom) diluted at 1:4,000 in a ready-to-use antibody diluent (Dako, Agilent Technologies, Carpinteria, CA) for 30 min at room temperature. This was followed by incubation with a polymer-labeled goat anti-rabbit IgG conjugated to HRP (8 min). 3,3′-Diaminobenzidine tetrahydrochloride (DAB) was used as the substrate, and the slides were incubated for 10 min. Tissue sections were subsequently incubated with a rat anti-mouse CD8a MAb (clone 4SM15; eBioscience, ThermoFisher Scientific, Waltham, MA) diluted at 1:1,600 in a ready-to-use antibody diluent (Dako, Agilent Technologies) for 30 min at room temperature. Tissue sections were subsequently incubated with a rabbit anti-rat IgG (Vector Labs, Burlingame, CA) diluted 1:1,000 for 30 min at room temperature, followed by a polymer-labeled goat anti-rabbit IgG coupled with alkaline phosphatase (30 min). Fast Red was used as the chromogen (15 min), and counterstaining was performed with hematoxylin. Slides were mounted with a permanent mounting medium (Micromount; Leica Biosystems). Mouse spleen sections were used as positive controls, and tissue sections not incubated with primary antibodies were used as negative controls, in addition to tissue sections where the second primary antibody (anti-CD8) was omitted. Immunostaining was semiquantitatively scored based on the cumulative number of positive cells in five high-magnification (40×) microscopic fields.

### *In vivo* T-cell depletion.

CD4^+^ and CD8^+^ T cells were depleted in mice by treatment with mouse anti-CD4 (clone GK1.5) and anti-CD8a (clone 2.43) MAbs, respectively. Rat IgG2b isotype control (clone LTF-2) was administered to the control group. All antibodies were all purchased from BioXCell, West Lebanon, NH. Mice were injected with 500 μg of the appropriate MAb intraperitoneally 1 day before and 2 days after B16F10n-1 tumor engraftment and continued with maintenance intraperitoneal injections of 250 μg of the appropriate MAbs every 5 days throughout the remainder of the experiment. All immune cell depletion was confirmed by flow cytometry.

### Flow cytometry analysis.

B16F10n-1 tumors were harvested at various time points and dissociated in gentleMACS C-tubes (Miltenyi Biotec, San Diego, CA) containing Hanks’ balanced salt solution (HBSS) buffer (ThermoFisher, Waltham, MA) using a gentleMACS Octo Dissociator (Miltenyi Biotec, San Diego, CA). For enzymatic digestion, type I collagenase from Clostridium histolyticum (0.1g/ml; ThermoFisher, Waltham, MA) and DNAse I from bovine pancreas (10 mg/ml; Sigma-Aldrich, St. Louis, MO) was added to each C-tube and incubated at 37°C for 30 min with constant shaking. The resulting dissociated cells were then filtered through a 70-μm-pore filter and subsequently washed with flow cytometry staining buffer (PBS with 0.5% fetal bovine serum) to get a single-cell suspension. To reduce nonspecific binding, cells were incubated with anti-mouse CD16/32 block (clone 2.4G2; Tonbo Biosciences) in the presence of Ghost Dye Red 780 (Tonbo Biosciences) at 4°C for 30 min. Subsequently, the blocked cells were washed and incubated for 30 min at 4°C with the following specific antibodies: fluorescein isothiocyanate (FITC)-conjugated anti-mouse CD3ε (clone 145-2C11; BioLegend), phycoerythrin (PE)-Cyanine7-conjugated anti-mouse CD4 (clone RM4-5; Tonbo Biosciences), allophycocyanin (APC)-conjugated anti-mouse CD8a (clone 53-6.7; Tonbo Biosciences), BV711-conjugated anti-mouse CD25 (clone PC61; BioLegend), and Alexa Fluor 700-conjugated anti-mouse CD45 (clone 13/2.3; Tonbo Biosciences). Following surface staining, cells were fixed and permeabilized using the Foxp3/Transcription Factor Staining Buffer set (eBioscience, San Diego, CA). After a 4°C overnight incubation in Foxp3 fixation/permeabilization buffer (eBioscience), cells were washed and resuspended in 1× permeabilization buffer (eBioscience) containing PE-conjugated anti-mouse Foxp3 MAb (FJK-16s; eBioscience) and incubated for 2 h at 4°C. Following staining, cells were washed prior to flow cytometric analysis using a BD LSRFortessa X-20 cell analyzer (BD Biosciences, San Jose, CA) and FlowJo software (TreeStar, Ashland, OR). Gating for various populations of cells was established based on “fluorescence minus one” (FMO) controls.

### Statistical analysis.

Data were analyzed using GraphPad Prism 8 software (GraphPad Software, Inc., San Diego, CA). Statistical analyses were performed by using the Student’s *t* test and one-way analysis of variance (ANOVA). Animal survival is presented using Kaplan-Meier survival curves, and rates were analyzed using a log rank (Mantel-Cox) test. Data are expressed as means ± standard error. *P* values of ≤0.05 were considered statistically significant.
